# Dose and time dependence of functional impairments in rat jejunum following ionizing radiation exposure

**DOI:** 10.14814/phy2.14960

**Published:** 2021-08-02

**Authors:** Alexandra A. Livanova, Arina A. Fedorova, Alexander V. Zavirsky, Anastasia E. Bikmurzina, Igor I. Krivoi, Alexander G. Markov

**Affiliations:** ^1^ Department of General Physiology St. Petersburg State University St. Petersburg Russia; ^2^ Department of Biology S.M. Kirov Military Medical Academy St. Petersburg Russia; ^3^ Department of Military Toxicology and Medical Defense S.M. Kirov Military Medical Academy St. Petersburg Russia

**Keywords:** ionizing radiation, jejunum, Ussing chamber

## Abstract

Ionizing radiation causes dramatic change in the transport and barrier functions of the intestine. The degree of radiation damage rate depends primarily on the absorbed dose and post‐irradiation time. Variety of experimental protocols providing different time points and doses exist, with the lack of a common approach. In this study, to develop a unified convenient experimental scheme, dose and time dependence of barrier and transport properties of rat jejunum following ionizing radiation exposure were examined. Male Wistar rats were exposed to total body X‐ray irradiation (2, 5, or 10 Gy). The control group was subjected to sham irradiation procedure. Samples of rat jejunum were obtained at 24, 48, or 72 h post‐irradiation. Transepithelial resistance, short circuit current (*I*_sc_), and paracellular permeability for sodium fluorescein of jejunum samples were measured in an Ussing chamber; a histological examination was also performed. These parameters were significantly disturbed only 72 h after irradiation at a dose of 10 Gy, which was accompanied by loss of crypt and villi, inflammatory infiltrations, and disintegration of enterocytes. This suggests that found experimental point (72 h after 10 Gy exposure) is the most appropriate for future study using rat jejunum as a model.

## Graphical abstract

Ionizing radiation has a severe impact on the barrier and transport functions of the intestine triggering acute gastrointestinal radiation syndrome and limiting the dose and effectiveness of radiation therapy. In this study, the electrophysiological characteristics and permeability of the jejunum of rats after X‐ray exposure were determined depending on the dose and the time post‐irradiation.

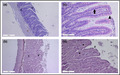

**New findings**

1 **What is the central question of this study?**

Ionizing radiation disturbs the gastrointestinal tract. Existing experimental protocols provide scattered data on the dose and time dependence of radiation effects with the lack of common approach. In this study, unified protocol is developed, using rat jejunum as a model.

2 **What is the main finding and its importance?**

Significant impairments of the structural and functional characteristics of the rat jejunum were shown 72 h after exposure of 10 Gy, which suggests that these conditions are most suitable for studying irradiation. Given the important role of the gastrointestinal tract in water–salt balance and in protection against bacterial intervention, these findings could be useful for future development of a new effective therapeutic strategy in radiation injury.

## 1 INTRODUCTION

Epithelium barrier and transport functions play a crucial role in maintenance of homeostasis. Epithelial barrier in the gastrointestinal tract provides protection against bacterial intervention as well as water and electrolyte balance support. Ionizing radiation causes damage to various tissues including gastrointestinal tract; radiation‐induced dysfunction of intestine is well documented (Dubois & Walker, 1988; Shadad et al., 2013). Gastrointestinal radiation injury is of great importance for several reasons. Intestine and colon lesion risks limit the dose of radiation under cancer radiotherapy, thus lowering the efficacy of treatment and sufficiently reduces patient's quality of life (Bacon et al., 2001; Sher, 2010). The danger of severe gastrointestinal impairment is also significant under high‐dose total body irradiation produced in case of nuclear power plant accidents and radiation incidents and must be considered when implementing a nuclear safety strategy. It is also documented that breaks in the gastrointestinal epithelial barrier are induced by space radiation exposure, with subsequent increase in translocation of bacterial products being the factor affecting astronauts' health (Kennedy, 2014; Ni et al., 2011; Zhou et al., 2012).

Radiation impairment is manifested in a tissue‐dependent manner and the colon is relatively resistant while the small intestine is more sensitive to radiation (Shukla at al., 2016). In addition, the microbiome of the small intestine is known to undergo significant alteration when exposed to ionizing radiation (Walker et al., 1985). Importantly, the mucosal microbiome is involved in various signaling pathways that link it to other body systems, including the immune, endocrine, and nervous systems (Jones et al., 2020). There is a lot of scattered data describing the molecular basis of physiological violation of the small intestine. Previous study showed that radiation disrupts intestinal epithelial tight and adherens junctions, as well as actin cytoskeleton (Shukla at al., 2016). Na,K‐ATPase, which plays a critical role in polarity and vectorial transport across epithelial cells and functionally interacts with claudins (Markov et al., 2020), is also a radiation target (Huang et al., 2019). The ability of circulating ouabain, a specific ligand of the Na,K‐ATPase, to affect claudins expression and therefore epithelial barrier properties, is also most pronounced in the jejunum (Markov et al., 2020). These observations suggest that jejunum can serve as a convenient model for studying the molecular mechanisms of radiation‐induced disorders and approaches to their prevention.

The degree of radiation damage will depend upon a variety of conditions, primarily on the absorbed dose and post‐irradiation time (Shadad et al., 2013). Variety of experimental protocols providing different time points and doses are used to explore radiation impact on gastrointestinal tract (Gunter‐Smith et al., 1986; Verheye‐Dua & Bohm, 2000; Yin et al., 2014). The lack of a common protocol in such studies is a significant drawback. In this study, to develop a unified convenient approach, dose and time dependence of barrier and transport properties of rat jejunum following ionizing radiation exposure were examined. As LD50/30 of X‐rays for rats was found to be 6.6 Gy (Challapalli et al., 2015), three doses were chosen for this study, characterizing the nonlethal (2 Gy), half‐lethal (5 Gy), and lethal (10 Gy) ranges.

## 2 MATERIALS AND METHODS

### 2.1 Ethical approval

Adult male Wistar rats (*Rattus norvegicus*; 350–400 g, Saint‐Petersburg State University vivarium, Russia, *N* = 20) were used in the experiments. An experimental protocol was approved by Saint‐Petersburg State University Ethics Committee for Animal Research (approval form reference number 131‐03‐3). The experimental protocol was in accordance with the EU requirements described in 2010/63/EU Directive on animal experiments. Animals were kept in a vivarium with 12:12 h light‐dark cycle and *ad libitum* access to food and water.

### 2.2 Animal irradiation

Rats were exposed to total body X‐ray irradiation (2, 5, or, 10 Gy) using RUM‐17 apparatus (MosRentgen). During the procedure, the animals were placed in a closed plexiglass box, which completely restricted their movement (the lid was equipped with holes for air conditioning). The focal length to the X‐ray tube was 50 cm, with the dose rate being 0.31 Gy/min. To check the absorbed dose, an individual dosimeter was used, followed by result interpretation with the use of GO‐32 measuring device (Spetsoborona). The control group was subjected to sham irradiation procedure in which the animals were placed in a box under a switched off X‐ray tube.

### 2.3 Animal anesthesia and euthanasia

Light anesthesia was induced by intramuscular injection of Zoletil 100 (tiletamine/zolazepam; Virbac) at a dose of 10 mg/kg followed by neck dislocation euthanasia.

### 2.4 Measurement of electrophysiological characteristics and paracellular permeability of the jejunum in an Ussing chamber

Samples of rat jejunum were obtained at 24, 48, or 72 h post‐irradiation. To estimate barrier properties of the epithelium (transepithelial resistance, TER), as well as passive and active transport through the epithelium (short circuit current, *I*_sc_), the Ussing chamber was used. Jejunum segments were placed in Ussing chambers filled with Krebs‐Ringer solution containing (in mM): NaCl, 119; KCl, 5; CaCl_2_, 1.2; MgCl_2_, 1.2; NaHCO_3_, 25; Na_2_HPO_4_, 1.6; NaH_2_PO_4_, 0.4; d‐glucose, 10 (pH 7.4). During recording (60 min), the solution was gassed with 95% O_2_ and 5% CO_2_ and heated to 37°C. The registration of *I*_sc_ was carried out when the voltage was fixed at zero level (0 mV). To determine TER, the voltage value was recorded when the current was fixed at 10 μA. TER was calculated according to Ohm's law, taking into account the area of the chamber aperture (0.126 cm²), and expressed in Ω cm^2^. Within 60 min of registration in the Ussing chamber, TER and *I*_sc_ values of the tissue remained stable for all samples. Therefore, TER and *I*_sc_ obtained at the 30th min of registration in the Ussing chamber are shown. To measure the paracellular permeability of the jejunum, 50 μl of sodium fluorescein (Sigma Aldrich) was added in the Ussing chamber from the apical side. The solution from the basolateral side was collected after 60 min of incubation and the concentration of sodium fluorescein was analyzed using a Typhoon FLA 9500 laser scanner (GE Healthcare).

### 2.5 Histological examination

Jejunum sections obtained from two groups of rats (sham irradiated and 10 Gy irradiated) were fixed in 10% formalin (BioVitrum) for 48 h and embedded in paraffin blocks. Five‐micrometer thick slides were sectioned using a rotary microtome Leica RM2265 (Leica Microsystems), and stained with hematoxylin and eosin. The jejunum tissues were examined using a Leica DMI6000 microscope (Leica Microsystems). Images were captured with digital camera (Leica) at ×100 and ×200 magnifications and analyzed with Leica Application Suite software (Leica Microsystems).

### 2.6 Statistical analysis

Statistical analysis was performed using Prism 8 software (GraphPad). The statistical significance of the means difference was assessed using two‐way analysis of variance (ANOVA). Dunnett's multiple comparisons test was used to compare groups of animals irradiated at different doses with the matched sham‐irradiated control group. Tukey's multiple comparisons test was used to compare subgroups of animals irradiated with a single dose at 24, 48, or 72 h post‐irradiation. All values shown are presented as mean ± SD. Data on every tissue segments placed in Ussing chamber was taken as an independent event. *p*‐values below 0.05 were considered statistically significant.

## 3 RESULTS

### 3.1 Transepithelial resistance

**FIGURE 1:**
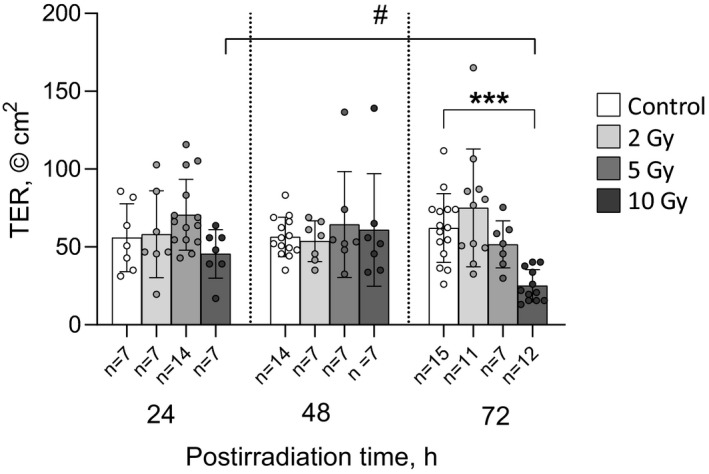
Transepithelial resistance (TER) of rat jejunum fragments after exposure to X‐ray irradiation at different doses with various post‐irradiation periods. The number of symbols corresponds to the number of fragments (*n*). Data are represented as means ± SD. ***p < 0.001—significant difference from matched control group, two‐way ANOVA with Dunnett's multiple comparisons test; ^#^ *p* < 0.05—significant difference between groups irradiated at single dose of 72 Gy and studied at different post‐irradiation periods (as indicated by horizontal bar), two‐way ANOVA with Tukey's multiple comparisons test

After 2 and 5 Gy exposure, TER did not significantly differ from matched controls at any post‐irradiation time (Figure 1). After 10 Gy intervention, TER was significantly reduced in comparison with matched control only at 72 h after irradiation (25.0 ± 10.3 and 62.1 ± 22.1 Ω cm^2^, respectively, *p* < 0.001) (Figure 1). TER values in the control groups, as well as in the groups of rats exposed at doses of 2 and 5 Gy, did not differ significantly after 24, 48, or 72 h post‐irradiation. In the group of animals irradiated at a dose of 10 Gy, TER was significantly lower 72 h after irradiation than that after 24 h (25.0 ± 10.3 and 45.5 ± 15.6 Ω cm^2^, respectively, *p* < 0.05; Figure 1).

### 3.2 Short circuit current (*I*_sc_)

**FIGURE 2:**
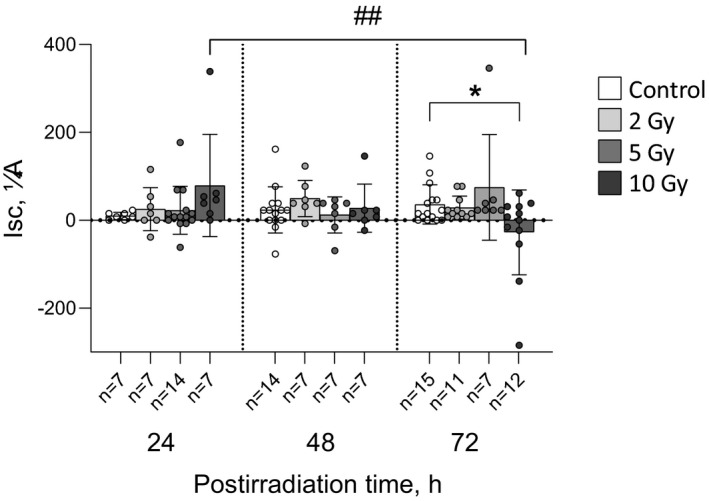
Short circuit current (*I*_sc_) of rat jejunum epithelium fragments after exposure to X‐ray irradiation at different doses with various post‐irradiation periods. The number of symbols corresponds to the number of fragments (*n*). Data are represented as means ± SD. *p < 0.05—significant difference from matched control group, two‐way ANOVA with Dunnett's multiple comparisons test; ^##^ *p* < 0.01—significant difference between groups irradiated at single dose of 72 Gy and studied at different post‐irradiation periods (as indicated by horizontal bar), two‐way ANOVA with Tukey's multiple comparisons test

After 2 and 5 Gy exposure, *I*_sc_ did not significantly differ from matched controls at any post‐irradiation time (Figure 2). After 10 Gy intervention, *I*_sc_ was significantly reduced in comparison with matched control only at 72 h after irradiation (−27 ± 96 and 36 ± 44 μA, respectively, *p* < 0.05; Figure 2). It should also be noted that in the group of 10‐Gy‐irradiated animals value of *I*_sc_ changed sign to negative by the 72nd hour post‐exposure (Figure 2). When comparing subgroups irradiated at single dose and observed at different time post‐exposure, difference between animals investigated at 72 and 24 h was found in the group exposed to 10 Gy irradiation (−27 ± 96 and 79 ± 116 μA, respectively, *p* < 0.01).

### 3.3 Permeability for sodium fluorescein

**FIGURE 3:**
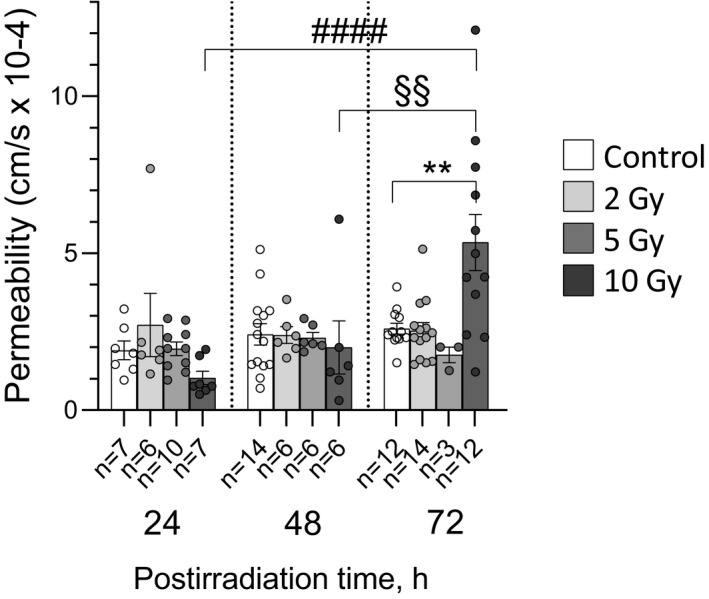
Permeability for sodium fluorescein of rat jejunum fragments after exposure to X‐ray irradiation at different doses with various post‐irradiation periods. The number of symbols corresponds to the number of fragments (*n*). Data are represented as means ± SD **p < 0.01—significant difference from matched control group, two‐way ANOVA with Dunnett's multiple comparisons test; ^####^ *p *< 0.001, ^§§^ *p* < 0.01—significant difference between groups irradiated at single dose of 72 Gy and studied at different post‐irradiation periods (as indicated by horizontal bar), two‐way with Tukey's multiple comparisons test

Simultaneous TER decrease and *I_sc_* increase (10 Gy, 72 h post‐irradiation) was accompanied with significant increase in the permeability for sodium fluorescein in comparison with matched control (5.3 ± 3.1 and 2.6 ± 0.6 cm/s × 10^−4^, respectively, *p* < 0.01; Figure 3). The permeability in the control groups, as well as in the groups of rats irradiated at doses of 2 and 5 Gy, did not differ significantly after 24, 48, or 72 h post‐exposure. Nevertheless, after 10 Gy intervention, the permeability increased in a time‐dependent manner by the 72nd hour post‐irradiation (Figure 3).

### 3.4 Histological examination

**FIGURE 4:**
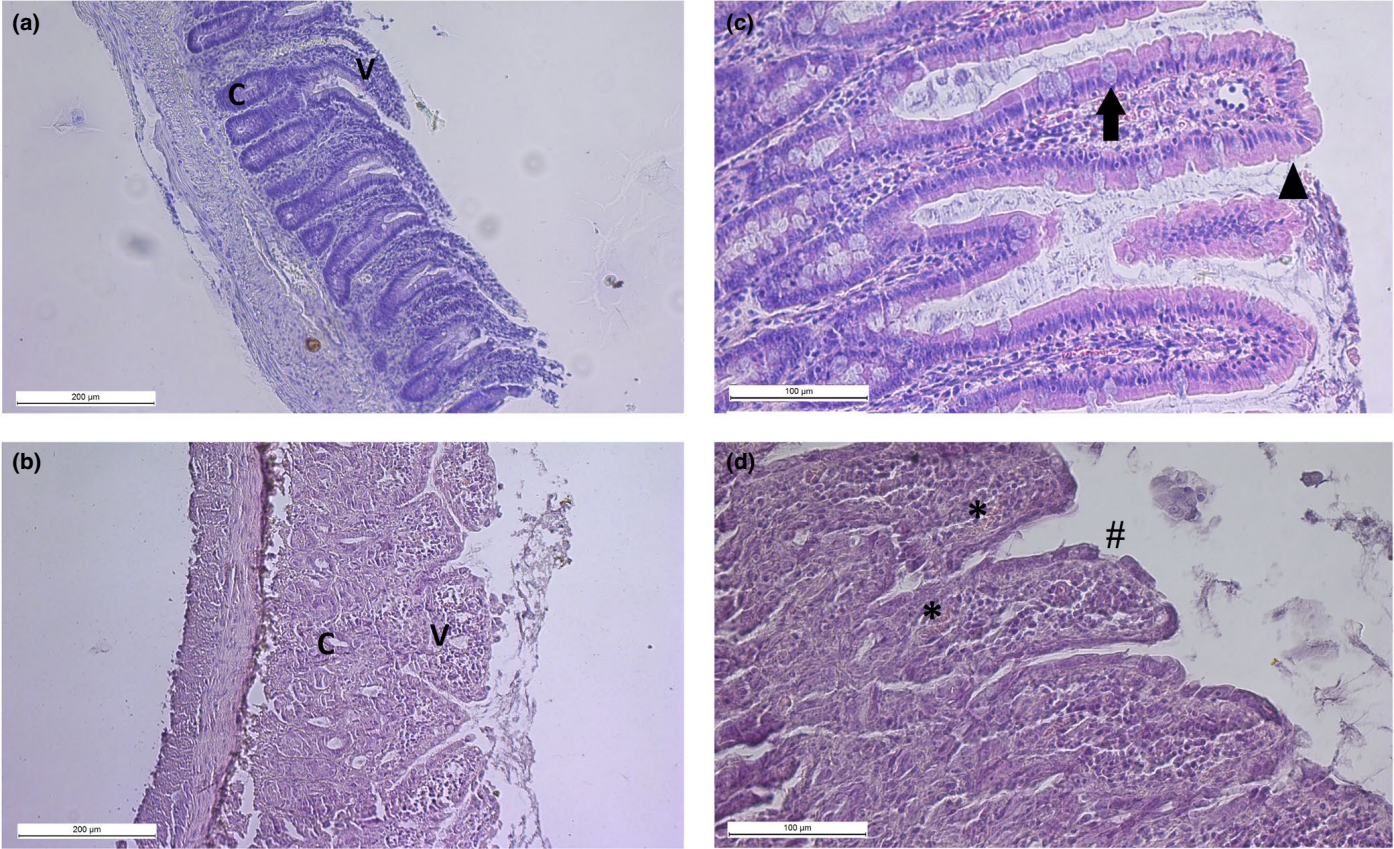
Histological micrographs of rat jejunum sections, hematoxylin and eosin staining. (a, ×100) and (b, ×200) represent sham irradiated group: typical histological structure of intestinal tissue with villi (V) and crypts (C) is observed. Brash border epithelium (arrowhead) and goblet cells (arrow) are monitored along the villi. (c, ×100) and (d, ×200) represent group irradiated at dose 10 Gy: the number of villi and crypts is decreased, villi are infused. Infiltration areas (asterisk) and disintegration of enterocytes (hash‐sign) are observed

Jejunum sections from sham‐irradiated control animals exhibited a typical histological structure of intestinal tissues with well‐defined villi and crypts (Figure 4a,b). Regular *lamina propria*, typical brush border epithelium, and numerous goblet cells were observed in control group. In sections obtained from the 10‐Gy‐irradiated group a decrease in intestinal villi number and height is determined, as well as fusion of intestinal villi, reduction of goblet cells, crypt loss, and disintegration of enterocytes (Figure 4c,d). Diffuse infiltration areas were also monitored in *lamina propria* in the 10‐Gy group.

## 4 DISCUSSION

Ionizing exposure significantly affects gastrointestinal tract with subsequent development of severe radiation injury (Garau et al., 2011). These molecular and cellular interventions are followed by impairments of physiological functions of intestinal epithelium (Shadad et al., 2013). To assess intestinal barrier and transport functions, a widespread electrophysiological method (Ussing chamber) is used, which makes it possible to investigate the ion transport (short circuit current, *I*_sc_) and the barrier properties of the epithelium (transepithelial resistance, TER; Thomson et al., 2019). Variety of protocols describing the examination of barrier and transport functions of the intestinal epithelium within an Ussing chamber is used. Earlier, within the study of rabbit ileum segments in Ussing chamber after the exposure to radiation at doses from 5 to 12 Gy at the appropriate time (1, 24, 48, 72, and 96 h), significant decrease in TER was observed, as well as a change in the values of *I*_sc_ (Gunter‐Smith et al., 1986). The mentioned study examined electrophysiological properties of the intestine when animals were exposed to high radiation doses causing severe gastrointestinal acute radiation syndrome. However, there are some data showing that ionizing radiation at lower doses also impacts functions of gastrointestinal tract (Casero et al., 2017; Liu et al., 2019). In mice, irradiated at doses of 1, 3, 5, and 7 Gy, an increase in intercellular sodium permeability of the ileum was revealed while studied in Ussing chamber (Yin et al., 2014). However, in this case, intestine post‐irradiation reactions were studied at only one period of time, namely, 6 days after exposure. Thus, a comprehensive analysis of small intestine barrier and transport functions at several periods of time elapsed after radiation exposure at different doses has not been carried out. In this paper, for the first time, a comparative analysis of electrophysiological parameters of rat jejunum epithelium 24, 48, and 72 h after total body irradiation with wide dose range is presented.

It was found that alterations in rat jejunum barrier and transport functions after total body irradiation depend both on the dose and on the time elapsed after irradiation. The most significant difference from the matched control group was observed 72 h after 10 Gy irradiation. The observed decrease in TER was accompanied by simultaneous increase in the paracellular permeability for sodium fluorescein, which together indicates a lesion of epithelial barrier. A decrease in barrier properties can be explained by a violation of the integrity of the intestinal epithelium and/or an increase in paracellular transport. Tight junction protein complexes are known to be responsible for the selective paracellular transport of ions and molecules in intestinal epithelium. Particularly, claudin‐family tight junction proteins make major contribution in providing the barrier properties (Markov et al., 2015). It was previously shown that radiation exposure dose dependently reduces the level of intestinal claudin‐3, which determines barrier tightness and ensures the impermeability of the epithelium (Huang et al., 2019; Milatz et al., 2010). The current study does not assess claudin‐3, however, it is proposed to obtain the level and redistribution of tight junction proteins as part of further work using experimental point obtained during this approach. It is possible that alteration in molecular components of tight junctions causes a violation of intestinal barrier functions after irradiation exposure.

As *I*_sc_ reflects the intensity of ion transport through the epithelium, I_sc_ decrease and change in sign from positive to negative probably indicates a dramatic change in the vector of ion transport between the intestinal lumen and the internal environment observed by 72 h after 10 Gy irradiation. Na,K‐ATPase is known to establish a transmembrane gradient of sodium and potassium ions (Matchkov & Krivoi, 2016) and functionally interact with claudins (Markov et al., 2020). Earlier in proteomic studies, it was shown that the expression of the Atp1b3 gene, encoding one of the subunits of Na,K‐ATPase, is significantly modulated in ileum epithelium of mice when exposed to ionizing radiation (Huang et al., 2019). Changes in Na,K‐ATPase level and activity may contribute to alterations in the value of *I*_sc_ and disturbance of transport functions of the intestinal epithelium after irradiation.

A histological examination of the jejunum specimens was carried out to confirm that a severe malfunction of the intestine, determined by the disturbance of the epithelium integrity, was observed after 72 h of irradiation at a dose of 10 Gy. The disintegration of enterocytes observed in this group may confirm the defects of tight junctions that arise after irradiation and are accompanied by a violation of the barrier properties.

It was found that significant violations of the barrier and transport properties of the intestine were observed only with irradiation at a dose of 10 Gy, despite the documented fact that radiation in lower doses also has an effect on the body, including the gastrointestinal tract. Thus, after irradiation of rats at a dose of 2 Gy an imbalance in pro‐ and antioxidative processes was shown, and oxidative stress was formed in enterocytes of the rat small intestine (Garau et al., 2011; Khizhniak et al., 2013). When rodents were irradiated at a dose of 5 Gy, individual apoptotic cells were found along the “crypt‐villus” axis of the intestinal epithelium (Garau et al., 2011; Mercantepe et al., 2019). Nevertheless, these changes probably did not affect the functional state of the intestinal barrier, which did not reveal in electrophysiological characteristics and intestinal permeability under irradiation in doses less than 10 Gy in our study.

Experimental point after 10 Gy irradiation at 72 h post‐irradiation coincides with acute gastrointestinal radiation syndrome which occurs at doses between 6 and 15 Gy (Garau et al., 2011). It is observed that between 7 and 10 days after exposure diarrhea, severe gastrointestinal injury, dehydration, and electrolyte loss occur following the denudation of intestinal mucosa. Our results show that these processes initialize at 72 h post‐irradiation in Wistar rat jejunum and according to gastrointestinal radiation syndrome characteristics this effect is long‐term. When exposed to a dose of 10 Gy, Wistar rats are known to die within 7 days with severe radiation damage, expressed in anorexia, weight loss, profuse diarrhea, facial edema, and ruffled hair (Challapalli et al., 2015). This means that if an attempt was made to set an experiment 96 h after irradiation and even later, some of the animals from the 10 Gy group would probably die, experiencing severe suffering. When the effect was found 72 h after irradiation, it was decided not to carry out experiments 96 h after the procedure, including in order to comply with ethical standards for working with animals.

Taken together, our data provide the evidence to suggest that 72 h after ionizing radiation exposure at dose of 10 Gy is most appropriate protocol for future study using rat jejunum as a model. This experimental point can be used when exploring effects of irradiation within modeling acute gastrointestinal radiation syndrome. We suggest that the disturbance of barrier and transport properties of intestine observed may reflect tight junction remodeling and alterations in Na,K‐ATPase activity.

## CONFLICT OF INTEREST

The authors declare that they have no competing interests.

## AUTHOR CONTRIBUTION

Conception or design of the work: Alexander G. Markov, Igor I. Krivoi, and Alexandra A. Livanova. Acquisition, analysis, or interpretation of data for the work: Alexandra A. Livanova, Arina A. Fedorova, Anastasia E. Bikmurzina, Alexander V. Zavirsky, Igor I. Krivoi, and Alexander G. Markov. Drafting of the work or revising it critically for important intellectual content: Alexandra A. Livanova, Arina A. Fedorova, Anastasia E. Bikmurzina, Alexander V. Zavirsky, Igor I. Krivoi, and Alexander G. Markov. All authors approved the final version of the manuscript and agree to be accountable for all aspects of the work in ensuring that questions related to the accuracy and integrity of any part of the work are appropriately investigated and resolved. All persons designated as authors qualify for authorship, and all those who qualify for authorship are listed.

## Data Availability

All data are available upon reasonable request to the authors.
